# Scientific Issues Relevant to Setting Regulatory Criteria to Identify Endocrine-Disrupting Substances in the European Union

**DOI:** 10.1289/EHP217

**Published:** 2016-04-25

**Authors:** Rémy Slama, Jean-Pierre Bourguignon, Barbara Demeneix, Richard Ivell, Giancarlo Panzica, Andreas Kortenkamp, R. Thomas Zoeller

**Affiliations:** 1 Team of Environmental Epidemiology, IAB (Institute of Advanced Biosciences), Inserm, CNRS, University Grenoble-Alpes, IAB joint research center, Grenoble (La Tronche), France; 2 Pediatric Endocrinology, CHU Liège and Neuroendocrinology Unit, GIGA Neurosciences, University of Liège, Belgium; 3 Department RDDM, Muséum National d’Histoire Naturelle, UMR CNRS/MNHN 7221, Paris, France; 4 School of Biosciences, and; 5 School of Veterinary Medicine and Science, University of Nottingham, Nottingham, United Kingdom; 6 Department of Neuroscience, University of Torino, Turin, Italy; 7 Neuroscience Institute Cavalieri Ottolenghi (NICO), Orbassano, Italy; 8 Institute of Environment, Health and Societies, Brunel University London, Uxbridge, United Kingdom; 9 Department of Biology, University of Massachusetts, Amherst, Massachusetts, USA

## Abstract

**Background:**

Endocrine disruptors (EDs) are defined by the World Health Organization (WHO) as exogenous compounds or mixtures that alter function(s) of the endocrine system and consequently cause adverse effects in an intact organism, or its progeny, or (sub)populations. European regulations on pesticides, biocides, cosmetics, and industrial chemicals require the European Commission to establish scientific criteria to define EDs.

**Objectives:**

We address the scientific relevance of four options for the identification of EDs proposed by the European Commission.

**Discussion:**

Option 1, which does not define EDs and leads to using interim criteria unrelated to the WHO definition of EDs, is not relevant. Options 2 and 3 rely on the WHO definition of EDs, which is widely accepted by the scientific community, with option 3 introducing additional categories based on the strength of evidence (suspected EDs and endocrine-active substances). Option 4 adds potency to the WHO definition, as a decision criterion. We argue that potency is dependent on the adverse effect considered and is scientifically ambiguous, and note that potency is not used as a criterion to define other particularly hazardous substances such as carcinogens and reproductive toxicants. The use of potency requires a context that goes beyond hazard identification and corresponds to risk characterization, in which potency (or, more relevantly, the dose–response function) is combined with exposure levels.

**Conclusions:**

There is scientific agreement regarding the adequacy of the WHO definition of EDs. The potency concept is not relevant to the identification of particularly serious hazards such as EDs. As is common practice for carcinogens, mutagens, and reproductive toxicants, a multi-level classification of ED based on the WHO definition, and not considering potency, would be relevant (corresponding to option 3 proposed by the European Commission).

**Citation:**

Slama R, Bourguignon JP, Demeneix B, Ivell R, Panzica G, Kortenkamp A, Zoeller RT. 2016. Scientific issues relevant to setting regulatory criteria to identify endocrine disrupting substances in the European Union. Environ Health Perspect 124:1497–1503; http://dx.doi.org/10.1289/EHP217

## Introduction

The regulation of chemicals identifies specific classes of health hazards such as carcinogens, mutagens, and reprotoxicants. Endocrine disruptors (EDs) are a newer type of hazard identified by research. The World Health Organization (WHO) defined an ED as “an exogenous substance or mixture that alters the function(s) of the endocrine system and consequently causes adverse effects in an intact organism, or its progeny, or (sub)populations” (WHO/IPCS 2002). Following the first scientific reference to EDs (Colborn et al. 1993), a large body of research has considerably improved our understanding of their effects in wildlife and humans (e.g., Bergman et al. 2013; Braun et al. 2011; Delfosse et al. 2014; Frye et al. 2012; Heindel et al. 2015; Kortenkamp et al. 2011; Shelton et al. 2014; Warner et al. 2014; Woodruff et al. 2011).

In 1999, the European Union (EU) became the first major economy to develop a strategy for the regulation of EDs (European Commission 1999). Subsequently, EDs have been addressed in at least four acts of EU law: the water framework directive (European Parliament 2000), REACH (the European Regulation on Registration, Evaluation, Authorisation and Restriction of Chemicals) (European Parliament 2006), the Plant Protection Products Regulation (PPPR) (European Parliament 2009a), the Cosmetics Regulation (European Parliament 2009b), as well as the Biocidal Products Regulation (BPR) (European Parliament 2012). The two latter regulations required the European Commission to establish scientific criteria to identify substances with endocrine-disrupting properties before December 2013.

**Figure 1:**
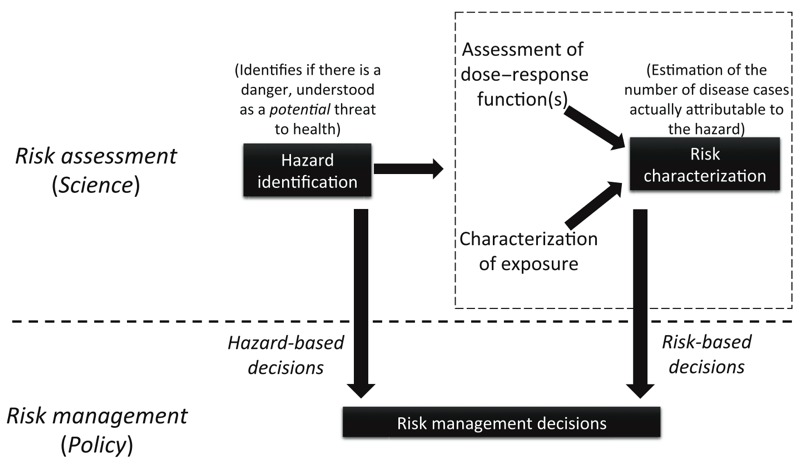
Hazard-based versus risk-based management of hazards. The step of risk characterization is sometimes (ambiguously) termed hazard characterization.

The PPPR and the BPR specify that substances with ED properties used as pesticides or biocides will not receive approval for their use, with certain exceptions (e.g., if exposure is negligible, for the PPPR). Similar provisions exist for carcinogens, mutagens, and reprotoxicants. Thus, these laws are not based on risk assessment for EDs present in biocides and pesticides, but only require hazard identification if exposure is not negligible. This corresponds to so-called “hazard-based cut-off criteria” (see Figure 1 for the distinction between hazard—a source of potential health effects—and risk—the actual impact of a substance in a population, in terms of disease probability or number of attributable disease cases). This hazard-based approach to pesticide and biocide regulation has been opposed by companies that market pesticides and biocides (CEFIC 2013; European Commission 2015; European Crop Protection Association 2014).

In addition, editors of pharmacology and toxicology journals condemned in an editorial the proposed European Commission recommendations on ED regulations, which they claimed were based on scientifically unfounded precaution and defied common sense and well-established risk assessment principles; the editors called for the consideration of adverse effects and potency (Dietrich et al. 2013). Their editorial was criticized for being based on a factually incorrect interpretation of the proposed regulatory framework and for ignoring the programming role of the endocrine system during development (Bergman et al. 2013; Gore et al. 2013). Its authors were also called upon to provide information about potential conflicts of interest (Grandjean and Ozonoff 2013).

At a meeting convened by the EU Commission including signatories of the Dietrich et al. (2013) editorial and scientists with a strong base in ED research, a consensus was reached on the definition of EDs, on the existence of nonmonotonic dose responses, and on the difficulties of determining thresholds for EDs (European Commission 2013).

Despite the obligations to establish scientific criteria to identify EDs by December 2013, as specified by EU laws (European Parliament 2009a, 2012), no such criteria have been published to date (April 2016) by the European Commission. Instead, the European Commission published a roadmap listing four options for defining criteria for identifying EDs and initiated an assessment of their impact (European Commission 2014) (Table 1). One of the options included in the roadmap (option 4) would use potency as a decision criterion during the process of hazard identification.

**Table 1:** Four options to identify endocrine-disrupting substances in the EC 2014 roadmap (European Commission 2014).

Option	Details	Comments
1	No criteria are specified. The interim criteria set in the BPR and PPPR continue to apply.	Would run counter to the PPPR and BPR, which require scientific criteria to be defined. Would lead to the interim criteria [which are not coherent with the WHO/IPCS (2002) definition of EDs] to be used.
2	WHO/IPCS definition (WHO/IPCS 2002) to identify ED (hazard identification). ED are identified as: a) Substances that are *i*) known or presumed to have caused endocrine-mediated adverse effects in humans or population-relevant endocrine-mediated adverse effects in animal species living in the environment or *ii*) where there is evidence from experimental studies (*in vivo*), possibly supported with other information (e.g., QSAR, analogue, and category approaches) to provide a strong presumption that the substance has the capacity to cause endocrine-mediated adverse effects in humans or population-relevant endocrine-mediated adverse effects on animal species living in the environment; b) The experimental studies used to determine if a substance is an endocrine disruptor shall provide clear evidence of endocrine-mediated adverse effects in the absence of other toxic effects, or, if occurring together with other toxic effects, the endocrine-mediated adverse effects should not be a nonspecific secondary consequence of other toxic effects; c) An adverse effect is a change in the morphology, physiology, growth, development, reproduction, or life span of an organism, system, or (sub)population that results in an impairment of functional capacity, an impairment of the capacity to compensate for additional stress, or an increase in susceptibility to other influences, as stated by WHO/IPCS (2009); d) where there is (e.g., mechanistic) information demonstrating that the effects are clearly not relevant for humans and not relevant at population level to animal species living in the environment, then the substance should not be considered an endocrine disruptor; e) The identification shall follow a step-by-step procedure as follows: *i*) gather all available data; *ii*) assess the data quality, reliability, reproducibility, and consistency; *iii*) consider adversity and mode of action together in a weight-of-evidence approach based on expert judgment; *iv*) evaluate whether endocrine disruption is attributable to a specific endocrine-mediated mode of action and not to a nonspecific secondary consequence of other toxic effects; *v*) evaluate human and wildlife relevance; *vi*) final (eco)toxicological evaluation indicating, where possible, whether the adverse effect is in relation to human health or environment (vertebrates and/or invertebrate populations), and where possible which are the axes or mechanisms concerned (e.g., estrogenic, androgenic, thyroid, and/or steroidogenic axes).	
3	WHO/IPCS (2002) definition to identify ED (hazard identification) as in Option 2. Introduction of additional categories based on the different strength of evidence for fulfilling the WHO/IPCS definition: Category I: “endocrine disruptors” (as defined in 2a–2d). Category II: “suspected endocrine disruptors,” defined as substances where there is some evidence for endocrine-mediated adverse effects from humans, animal species living in the environment, or experimental studies, but where the evidence is not sufficiently strong to place the substance in Category I. If, for example, limitations in the studies make the quality of evidence less convincing, Category II could be more appropriate. Points 2b, 2c (definition of adverse effect), and 2d above remain valid for Category II. Category III: “endocrine-active substances,” defined as substances for which there is some *in vitro* or *in vivo* evidence indicating a potential for endocrine disruption–mediated adverse effects in intact organisms and where the evidence is not sufficiently convincing to place the substance in Category I or II. The allocation to categories shall follow a step-by-step procedure (identical to that listed in 2e above).	The definition of “endocrine-active substances” (Category III) does not follow the definition provided by EFSA, which refers to substances that can interfere or react with the endocrine system (without evidence of adverse effect).
4	WHO/IPCS definition (WHO/IPCS 2002) to identify ED (hazard identification) and inclusion of potency as element of hazard characterization.	Potency is not defined. Option 4 introduces elements of risk assessment. No step-by-step procedure provided as in 2 and 3.
Abbreviations: BPR, Biocide Products Regulation (EU); PPPR, Plant Protection Products Regulation (EU); QSAR, quantitative structure–activity relationship.		

The disregard for the obligations laid down in EU law led Sweden and several other EU countries to sue the European Commission. In December 2015, the European Court of Justice ruled that the European Commission acted unlawfully in failing to develop ED criteria and that an impact assessment was unnecessary (European Court of Justice 2015). This judgment heightened the urgency of developing scientifically-based regulatory criteria for identifying EDs.

We elaborate some principles of ED regulation and specifically discuss the scientific relevance of each option considered by the European Commission to identify an ED, reviewing the availability of accepted definitions of EDs, endocrine-active substances, and the relevance of the concept of potency for hazard identification. A parallel with carcinogens is drawn. The relevance of impact assessment studies to define scientific criteria is finally discussed.

## Discussion

### Proposed Options Regarding Criteria for EDs in Europe

The general intention of defining ED criteria is “to ensure a high level of protection to human health and the environment and to strengthen the functioning of the internal market” (European Commission 2014). The four options proposed (European Commission 2014) are detailed in Table 1 and summarized below:

Option 1 consists of no policy change and no specification of criteria.
Option 2 relies on the WHO definition to identify EDs (WHO/IPCS 2002). This option *a*) identifies EDs as substances known or presumed to cause endocrine-mediated adverse effects in humans or animal species living in the environment; *b*) stipulates that endocrine-mediated adverse effects should not be a nonspecific secondary consequence of other toxic effects; *c*) defines “adverse effects” (as discussed below); *d*) excludes substances for which there is information demonstrating that the effects are not relevant for humans and for animal species living in the environment; and finally *e*) lists the step-by-step procedure to be followed for the identification.
Option 3 relies on the identification of ED as in Option 2 and further defines “suspected endocrine disruptors” and “endocrine active substances” (see below).
Option 4 relies on the WHO/IPCS definition of ED, and includes “potency” as an element of hazard characterization. Potency is not defined, nor is the manner in which it would be combined with the ED definition.

The European Commission (2014) indicated that Option 1 (no specification of criteria) would run counter to the requirements of regulations calling for an operational definition of EDs. Moreover, the PPPR and BPR laws mention interim criteria, and these would likely apply. According to these interim criteria, all substances classified as carcinogenic category 2 and toxic for reproduction category 2 shall be considered as EDs (European Parliament 2009a). These interim criteria based on the definitions of carcinogens and reproductive toxicants have no scientific relevance to the WHO/IPCS definition of endocrine disruptors (WHO/IPCS 2002), so Option 1 would not be scientifically justified. Consequently, we do not discuss this option further.

### Availability of a Definition of EDs

Option 2 of the roadmap defines EDs and adverse effect. At a workshop convened in 1996 in Weybridge (UK) by the European Commission, WHO and other institutions, an ED was defined as “an exogenous substance that causes adverse health effects in an intact organism, or its progeny, secondary to changes in endocrine function” (quoted by EFSA Scientific Committee 2013). Several definitions were subsequently suggested by Canadian, Japanese, and other institutions (reviewed by Kortenkamp et al. 2011), after which the International Program on Chemical Safety (IPCS), in collaboration with experts from Canada, Japan, the United States, and the EU, defined an ED as “an exogenous substance or mixture that alters the function(s) of the endocrine system and consequently causes adverse effects in an intact organism, or its progeny, or (sub)populations” (WHO/IPCS 2002). The main differences from the Weybridge definition are the consideration of mixtures and of effects in populations or subpopulations.

The definition issued from the workshop convened by the U.S. Environmental Protection Agency (EPA) in 1995 in Raleigh, North Carolina (Kavlock et al. 1996), which is still referred to by the U.S. EPA (2015), differs from the WHO/IPCS definition by lack of reference to adverse effects. As discussed below, substances acting on the endocrine system without evidence of an adverse health effect would be defined as endocrine-active substances under Option 3.

For other categories of health hazards, specific adverse health effects are often referred to, as is the case for carcinogens or reprotoxins, whereas for mutagens there is only a reference to a mode of action. The WHO/IPCS definition of EDs refers to both a mode of action and an adverse effect at the scale of organs, organisms, or populations. Consequently, conclusions about the nature of an ED require the integration of biochemical, toxicological, and ecotoxicological/human data.

EFSA (European Food Safety Authority) recommended that the WHO/IPCS definition be “adopted as a basis for the criteria for the identification of EDs” (EFSA Scientific Committee 2013). The European Commission roadmap acknowledges that “there is general consensus on the WHO/IPCS (2002) definition of an ED” (European Commission 2014).

The ED definition mentions “adverse effects.” Adverse effects were defined as a “change in the morphology, physiology, growth, development, reproduction or lifespan of an organism, system or (sub)population that results in an impairment of functional capacity, an impairment of the capacity to compensate for additional stress or an increase in susceptibility to other influences” (WHO/IPCS 2009). The EC roadmap explicitly refers to this definition. This definition covers health effects at the individual level such as occurrence of diabetes or obesity, IQ loss, as well as congenital malformations, or changes not visible at the individual but only at the population level, such as alteration of the sex ratio. It excludes, among others, transient changes in hormone levels that would not induce health effects in the short or long term. To our knowledge, this definition has not been questioned. The expression of “(sub)population” in WHO/IPCS definition refers to effects that may concern the population as a whole or a specific subgroup (e.g., based on sex, age, genetic susceptibility).

### Suspected EDs and Endocrine Active Substances (Option 3)

In addition to defining an ED as in Option 2, Option 3 proposes two additional categories, suspected endocrine disruptors and endocrine-active substances, that express the strength of evidence for a given compound.

“Suspected endocrine disruptors” are defined in the roadmap as “Substances where there is some evidence for endocrine-mediated adverse effects from humans, animal species living in the environment or from experimental studies, but where the evidence is not sufficiently strong to place the substance in Category I” (European Commission 2014). This definition is close to the WHO/IPCS definition of a “possible endocrine disruptor” (“an exogenous substance or mixture that possesses properties that might be expected to lead to endocrine disruption in an intact organism, or its progeny, or (sub)populations”) (WHO/IPCS 2002). “Endocrine-active substances” are defined in the European Commission roadmap as “Substances for which there is some … potential for endocrine disruption mediated adverse effects in intact organisms and where the evidence is not sufficiently convincing to place the substance in category I [ED] or II [suspected ED]” (European Commission 2014). We believe that the terminology of “endocrine-active substance” does not convey this lower level of evidence (a hierarchy such as ED [category I], presumed ED and suspected ED, similar to that of carcinogens shown in Table 2, would better fit this purpose). In contrast, an “endocrine-active substance” is defined by EFSA as “any chemical that can interact directly or indirectly with the endocrine system, and subsequently result in an effect on the endocrine system, target organs and tissues” (EFSA Scientific Committee 2013). The term is used to cover “all substances that in some way interfere with the endocrine system, but need not necessarily induce adverse effects.” This definition transmits the notion that there is evidence regarding the mode of action of the substance (interference with the endocrine system), but not regarding the induction of adverse effects, which is in line with the terminology of endocrine-active substances. Therefore, we suggest using the EFSA definition for endocrine-active substances instead of the EC roadmap definition.

**Table 2:** Categories of carcinogenic substances, as defined by the EU CLP regulation (EC, No. 1272/2008 on classification, labeling and packaging of substances and mixtures).

Carcinogens^*a*^		Endocrine-disrupting chemicals (Option 3 of the EC roadmap)	
Hazard class		Hazard class
Category Ia	Substances known to have carcinogenic potential for humans^*b*^	I	Substances known or presumed to be an endocrine disruptor
Category Ib	Substances presumed to have carcinogenic potential for humans^*b*^	II	Suspected endocrine disruptors
Category II	Suspected human carcinogens^*c*^	III	Endocrine-active substances
In the right-hand column, we have added the 3 levels for EDs proposed in Option 3 of the European Commission (2014) roadmap. ^***a***^A carcinogen is defined as a substance or a mixture of substances which induce cancer or increase its incidence. Substances that have induced benign and malignant tumors in well-performed experimental studies on animals are considered also to be presumed or suspected human carcinogens unless there is strong evidence that the mechanism of tumor formation is not relevant for humans (European Parliament 2008). ^***b***^A substance is classified in Category I for carcinogenicity on the basis of epidemiological and/or animal data. A substance may be further distinguished as Category IA, known to have carcinogenic potential for humans, classification largely based on human evidence, or Category IB, presumed to have carcinogenic potential for humans, classification largely based on animal evidence. ^***c***^According to the EU regulation, the placing of a substance in Category II (suspected human carcinogens) is done on the basis of evidence obtained from human and/or animal studies, but which is not sufficiently convincing to place the substance in Category IA or IB, based on strength of evidence together with additional considerations. Such evidence may be derived either from limited evidence of carcinogenicity in human studies or from limited evidence of carcinogenicity in animal studies.			

### Introduction of Potency as a Criterion for Hazard Identification (Option 4)

Option 4 of the roadmap is based on the WHO/IPCS definition of an ED, with potency as an added criterion. This option echoes approaches developed by the United Kingdom and German authorities with the explicit intention of limiting the number of substances that would fall under the hazard-based cut-off criteria of the PPPR and BPR (discussed by Kortenkamp et al. 2011). A publication from the German federal institute for risk assessment also suggested to consider potency to identify EDs (Marx-Stoelting et al. 2015).

Potency is not well defined; it is not in the glossary of terms of the environmental health criteria published by the International Program on Chemical Safety (IPCS 2009). The term is presented in a publication sponsored by ECETOC—the European Centre for Ecotoxicology and Toxicology of Chemicals, a nonprofit association of companies with interests in the manufacture and use of chemicals—as being “primarily based on the dose causing a specific toxic effect” without being clearly defined (Hennes et al. 2014). A publication from the German federal institute for risk assessment indicates that “Potency relates to the dose levels at which certain effects occur” (Marx-Stoelting et al. 2015). The International Union of Pharmacology defines potency as “an expression of the activity of a drug, in terms of the concentration or amount needed to produce a defined effect; an imprecise term that should always be further defined (see EC_50_, IC_50_, etc.),” where EC_50_ is further defined as “The molar concentration of an agonist that produces 50% of the maximal possible effect of that agonist. Other percentage values (EC_20_, EC_40_, etc.) can be specified” (Neubig et al. 2003).

**Figure 2:**
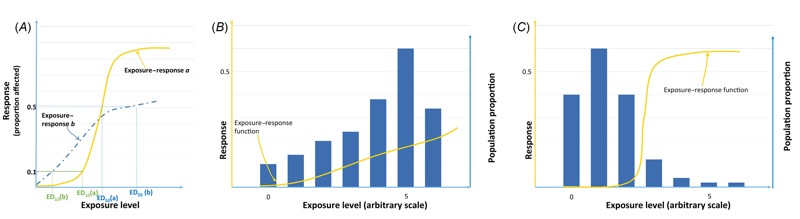
Illustration of issues with the potency concept, with hypothetical dose–response functions and distributions of exposure. (*A*) Situation of dose–response functions that cross: If potency is defined as the dose ED_50_ leading to 50% of a given response, then chemical with the dose–response function *a* is considered more potent than chemical with exposure–response function *b*; if potency is defined as the dose leading to 10% of the response (ED_10_), then chemical with dose–response function *a* is less potent than chemical with dose–response function *b*. (*B*) Shallow dose–response function (and low potency) with a large proportion of highly exposed subjects, hence entailing a possibly high risk. (*C*) Steep dose–response function (and high potency) with a low proportion of highly exposed subjects, hence entailing a possibly similar or lower risk. Blue bars in *B* and *C* represent the distribution of exposure in the population.

Hence, in pharmacology, potency is related to the dose–response function: A substance that at a certain dose causes 50% of its possible maximal effect magnitude (e.g., rate of animals with a specific disease) is considered more potent than another substance for which the same effect magnitude is attained at a larger dose. As already mentioned (Neubig et al. 2003), sometimes doses other than those leading to 50% of a given effect are used, such as 10% of a given effect, without apparent scientific justification of how these cut-off values are chosen. Thus, potency is simply a point of the dose–response function, corresponding to the dose at which this dose–response function intersects an arbitrary response level (Figure 2A).

The step-by-step procedure of the EC roadmap (Options 2 and 3) mentions that it is necessary to evaluate whether endocrine disruption is attributable to a specific endocrine-mediated mode of action and not to a nonspecific secondary consequence of other toxic effects (European Commission 2014). Consequently, effects that would occur at very high doses at which general toxicity is observed would not be enough to qualify the compound as an ED, without the need to explicitly introduce concepts related to the dose at which effects occur.

The introduction of potency as a criterion in hazard identification would lead to several difficulties. First, this concept is not suited for compounds for which nonmonotonic dose–response functions are possible, as is the case for EDs (Vandenberg et al. 2012). Second, the introduction of potency as a decision criterion may force the establishment of dichotomous regulatory cut-off values that are entirely arbitrary and not science-based, such that a compound with a potency of 10 mg/kg/day might be classified as an ED, while a compound with a potency of 11 mg/kg/day (hence causing the same effect at an exposure of 11 instead of 10 mg/kg/day) would not be classified as an ED. Third, potency comparisons are influenced by the effect magnitude that is chosen to define the doses to be compared (i.e., whether one considers a 10% or a 50% increase; see Figure 2A) and by the health end point considered to define potency. Overall, potency is not a relevant concept for hazard identification.

Even in the context of risk management, potency alone is of little use. Indeed, dose–response functions, from which potency is defined, are not meaningful alone and need to be interpreted in relation to exposure, which allows estimation of the level of risk for a given population (Figure 1). Low potency compounds with shallow dose–response functions and very frequent exposures (Figure 2B) may present greater risks at the population level than more potent chemicals with steep dose–response functions but less frequent exposure (Figure 2C). Well-established examples illustrating that the dose response (or potency) cannot be considered alone to predict risk include airborne fine particulate matter (≤ 2.5 μm; PM_2.5_) (WHO 2014) and low exposures during critical windows of vulnerability like fetal development, such as those demonstrated for effects of PCBs on intellectual quotient (Jacobson and Jacobson 1996; Schantz et al. 2003). Accordingly, the EFSA scientific committee stated “that, to assess whether or not a (predefined) level of concern is reached for an ED, potency should not be used alone but should take account of actual or predicted exposure” (EFSA Scientific Committee 2013). Indeed, potency replaces dose–response curves by a single point of the curve, which results in a strong loss of information. If a risk-based and not hazard-based management is chosen, the relevant approach is to take into account the variations of the dose–response function over the whole range of exposures and combine it with actual exposures, for all relevant health outcomes, in other words to explicitly perform a risk assessment study—but this goes beyond the steps required for hazard identification.

In the context of the PPPR, where some substances are to be regulated mostly on the basis of their hazard (at least if exposure is not negligible) and not their risk, considering dose–response functions (or potency) at the step of hazard identification would lead to reintroducing a logic of risk assessment. The discussion of whether or not the hazard-based logic of the PPPR and BPR for EDs should be modified into a risk-based regulation is a matter of policy. If deemed relevant by regulators, risk assessment should not be reintroduced partially (by considering only a component of risk assessment), nor “by the back door,” indirectly, by requiring consideration of a criterion related to risk assessment such as potency. Rather, if necessary, this should be done explicitly, by modifying the legislation.

### Parallel with Hazard Identification in the Field of Carcinogens

Another key argument against adopting criteria considering potency is consistency with the identification of other hazards of similar concern, such as carcinogens or reproductive toxicants. Several other types of chemical hazards are explicitly referred to in the EU regulation, including carcinogens, mutagens, reprotoxins. Carcinogens are defined as “a substance or a mixture of substances which induce cancer or increase its incidence. Substances which have induced benign and malignant tumors in well-performed experimental studies on animals are considered also to be presumed or suspected human carcinogens unless there is strong evidence that the mechanism of tumor formation is not relevant for humans” (European Parliament 2008). For carcinogens, the EU defines three categories for carcinogenic substances (Ia, Ib, and II, the last corresponding to suspected carcinogens, Table 2). The classification of a substance in any category is based on a scientific assessment of the hazard (hazard identification) and does not take into consideration other components of the risk assessment scheme (Figure 1) such as “potency.” Opting for options 2 or 4 would separate EDs from other hazards of equivalent concerns because the number of hazard categories would differ (in the case of Option 2, for which a substance is either identified as an ED or not, not alerting industry, consumers or policy makers to suspected EDs) or because potency would be considered (Option 4). This would run counter to the policy choice of the legislation to consider EDs as being of equivalent concern to carcinogens, mutagens, and reprotoxicants. Overall, the example of carcinogens shows that criteria defining a serious hazard need not be complex, nor need to resort to potency and risk-related concepts.

### Impact Assessment Studies Are Not Designed to Help Defining Hazards

The European Commission is carrying out an impact assessment as a preliminary step before deciding among the four options. Impact assessment studies provide an assessment of the potential economic, social and environmental impacts of alternative policy options. They would make sense if policy options were currently examined (e.g., between hazard-based regulation of pesticides or risk-based regulation), or after the implementation of a policy to judge its results. Here the relevant regulations (PPPR, BPR, REACH laws) have already been enacted but not applied as far as EDs are concerned.

Scientific criteria should rely on a scientific foundation. It is not the evaluation of the impact of a family of compounds that should guide their scientific definition; rather, the adoption of a scientific definition conditions any impact evaluation. Continuing the previous parallel with other health hazards, carcinogens were defined before obtaining a clear picture of the number of existing carcinogens, and independently of their impact. Similarly, it would not be necessary to perform an impact assessment study before defining X-rays or explosives.

Studies of the impact of some EDs on disease burden and cost in Europe have already been published (Trasande et al. 2015). The economic cost associated with exposure to non-banned EDs in the EU was estimated to be 157 billion euros per year (Trasande et al. 2015).

If option A leads to the identification of 10 substances that are EDs while option B identifies 50 further substances, will option B be preferred to limit the health impact of EDs or will option A be chosen to limit constraints on the industrial sector? Economic and health impacts are subject to quick changes as a function of exposure levels, development of substitutes or alternative industrial processes, and existence of companies with relevant substitutes. Will the impact assessment be updated to take these changes into account, and the criteria modified accordingly?

In its ruling against the European Commission, the European court of justice stated that “the definition of scientific criteria to identify properties disrupting the endocrine system can only be done in an objective manner based on scientific data relative to the endocrine system, independently from any other consideration, and in particular from any economic consideration” (European Court of Justice 2015). Making a scientific definition dependent on the results of an assessment of its impact would be a dangerous precedent for public health and science in general.

## Conclusion

The laws passed by the European parliament during the last decade constitute an innovative approach to limit health risks posed by EDs.

We have presented and discussed each option proposed by the European Commission to identify EDs (European Commission 2014), and provided specific recommendations (Table 3). Only Options 2 and 3 comply with science. There is scientific consensus on the relevance of the WHO/IPCS definition of an ED (WHO/IPCS 2002). Option 4 modifies this definition by introducing the notion of potency, which is absent from the WHO/IPCS definition and from the criteria identifying carcinogens, which are hazards of equivalent concern to EDs. We believe that, because of the parallel with definitions of carcinogenic hazards (which have different categories based on evidence levels) and because it calls for the identification of suspected EDs, Option 3 is more relevant. This will provide a simple classification conveying the weight of the scientific evidence regarding the likelihood for the compound to be an ED: endocrine disruptors (including substances known or presumed to be EDs), suspected endocrine disruptors, and endocrine-active substances (Table 2).

**Table 3:** Recommendations.

Recommendation	Rationale
1. Refer to the WHO/IPCS (2002) definition of EDs, potential (suspected) ED, and adverse effects; and to the EFSA definition of endocrine active substances.	Follow scientific consensus.
2. Identify hazards without referring to potency.	Potency is poorly defined and end point dependent, is not used to define other hazards of equivalent concern such as carcinogens, and belongs to risk assessment, not hazard identification.
3. Consider hazard identification and risk characterization as separate issues. Do not use scientific criteria to move from a hazard-based to a risk-based regulation for specific substances.	Any change in the spirit of the law should be done explicitly in the law, not via a delegate act.
4. Establish scientific ED criteria regardless of an impact assessment study.	Impact assessment studies are not meant to provide scientific definitions.
5. Incorporate the level of evidence in characterization of EDs (Option 3).	Proven to be relevant for carcinogens and other hazardous substances of equivalent concern to EDs.

We recognize that scientific uncertainty remains with regard to the finer detail of mechanisms, the exact extent of health and environmental effects of EDs, and their impact at the population level. There are also great uncertainties as to the number of substances likely to be identified as EDs. However, as demonstrated by the 40 years of work by the International Agency for Research on Cancer to identify carcinogens (Pearce et al. 2015), the availability of a clear definition of the hazard considered is a necessary first step. Once defining criteria are available, one can develop appropriate testing methods, identify substances, and manage risk. Some of the test methods that will be required for regulatory purposes need to be developed and agreed upon.

There is no scientific or public health justification for the delay in the adoption of scientific criteria for EDs.

As scientists, we believe that impact assessment studies should not be used to define scientific criteria, nor be used as an argument for postponing the publication of a scientific definition. We are concerned that an impact assessment study could be used to bend science toward an outcome defined by aspects external to science. We are convinced that the (vague) notion of potency has no place in a hazard identification context. We are concerned that scientific definitions are being distorted in order to modify the spirit of a law that requires hazard-based management of EDs present in pesticides if exposure is not negligible, and not a risk-based management, thereby muddling science and policy. We believe that scientific criteria identifying EDs should follow the logic of the EU criteria for other serious hazards such as carcinogens and reproductive toxicants. We regret that several years have been spent trying to issue scientific criteria defining a hazard that actually has been defined years earlier by a state-of-the-science report from WHO. We fear that the most plausible explanation for this delay is not a lack of scientific consensus, but rather that postponing the publication of the scientific criteria is a way to postpone the full application of the 2009 pesticide regulation and 2012 biocide European regulation. This postponement is all the more worrying because these scientific criteria are but one of the first steps toward identifying EDs and providing more efficient protection of public health in the European Union.

***Note added in proof:*** *After acceptance of this manuscript, on 16 June 2016, the European Commission published proposals to define EDs in the context of the pesticides and biocides regulations (* *European Commission 2016* *). The proposal suggested abandoning the hazard-based logic of management of pesticides containing EDs. See* *Kortenkamp et al. (2016)* *for a comment.*
